# Remaining Useful Life Prediction Model for Rolling Bearings Based on MFPE–MACNN

**DOI:** 10.3390/e24070905

**Published:** 2022-06-30

**Authors:** Yaping Wang, Jinbao Wang, Sheng Zhang, Di Xu, Jianghua Ge

**Affiliations:** 1 Key Laboratory of Advanced Manufacturing and Intelligent Technology of Ministry of Education, Harbin University of Science and Technology, Harbin 150080, China; wypbl@163.com (Y.W.); gejianghua0619@gmail.com (J.G.); 2 School of Mechanical and Power Engineering, Harbin University of Science and Technology, Harbin 150080, China; wang_1411@163.com (J.W.); cxzzxc147258@163.com (S.Z.)

**Keywords:** multiscale fusion permutation entropy, multiscale convolutional attention neural network, resonance sparse decomposition method, remaining useful life prediction, rolling bearing

## Abstract

Aiming to resolve the problem of redundant information concerning rolling bearing degradation characteristics and to tackle the difficulty faced by convolutional deep learning models in learning feature information in complex time series, a prediction model for remaining useful life based on multiscale fusion permutation entropy (MFPE) and a multiscale convolutional attention neural network (MACNN) is proposed. The original signal of the rolling bearing was extracted and decomposed by resonance sparse decomposition to obtain the high-resonance and low-resonance components. The multiscale permutation entropy of the low-resonance component was calculated. Moreover, the locally linear-embedding algorithm was used for dimensionality reduction to remove redundant information. The multiscale convolution module was constructed to learn the feature information at different time scales. The attention module was used to fuse the feature information and input it into the remaining useful life prediction module for evaluation. The appropriate network structure and parameter configuration were determined, and a multiscale convolutional attention neural network was designed to determine the remaining useful life prediction model. The results show that the method demonstrates effectiveness and superiority in degrading the feature information representation and improving the remaining useful life prediction accuracy compared with other models.

## 1. Introduction

With the rapid development of the Industrial Internet of Things, the explosive growth of monitoring data brings new opportunities and challenges for predictions of the remaining useful life of rolling bearings. The data-driven remaining useful life prediction method can learn the degradation characteristics of rolling bearings from the massive monitoring data and build a corresponding remaining useful life prediction model. Therefore, it has received increasing attention in research surrounding remaining useful life prediction [1].

Data-driven methods for remaining useful life prediction based on data typically involve three steps, including degradation feature construction, degradation trend learning, and remaining useful life estimation [2]. In the task of rolling bearing remaining useful life prediction, the trend of rolling bearing remaining useful life degradation over time needs to be better evaluated. Therefore, increasingly time-sensitive features need to be extracted. Degradation feature construction uses a priori knowledge of rolling bearing performance to extract sensitive degradation features from the monitoring data obtained. At the current stage, rolling bearing vibration signal feature-extraction methods mainly remove the signal features reflecting time, and remove the frequency domain waveform characteristics from signals from the time and frequency domains. The methods also utilize other basic processes, such as root mean square and kurtosis. Although these signal features can reflect the fault information in a bearing signal [3], they still have a problem: insensitivity to the trend of decline of rolling bearings over time. The resonance sparse decomposition method is a signal processing method proposed by Selesnick [4] in 2011. The periodic vibration components generated by the regular bearing operation, and the periodic shock component developed by the bearing failure, can correspond well to high-resonance and low-resonance components generated under the decomposition of bearing vibration signals by the resonance sparse decomposition algorithm. Compared with the signal enhancement method, based on the vibration signal spectrum, the resonance sparse decomposition algorithm can directly extract low-resonance components. These contain more fault information from the vibration signal, avoid the limitation of spectrum analysis, and are more suitable for processing nonlinear signals. Permutation entropy is a method proposed by Bandt et al. [5] to detect the randomness and kinetic mutation of time series, and it has good anti-transformation properties for mutated, non-smooth signals. Mengjie Liu et al. [6] demonstrated that permutation entropy has an excellent ability to characterize different faults occurring in rolling bearings by comparing the performance of permutation entropy, approximate entropy, and Lempel–Zi complexity in bearing fault diagnosis. However, permutation entropy can only evaluate the characteristic information of the signal from a rolling bearing vibration on a single time scale, which may cause the critical, distinct information to be insignificant. At this stage, the rolling bearing vibration signal is complex, and an evaluation only from a single time scale can no longer reflect its complete characteristic information. Ge et al. [7] proposed multiscale permutation entropy combined with robust principal component analysis (RPCA), which can reflect deeper features of the signal by setting different scale factors [8,9]. The diagnosis of bearing faults can effectively detect and locate bearing faults. Ye et al. [10] proposed a feature-extraction method, VMD-MPE. They demonstrated that MPE could represent the feature information of rolling bearings by comparing experiments with VMD-MSE, VMD-MFE, EMD-MPE, and WT-MPE. Du et al. [11] used MPE to extract fault features and combined it with a self-organizing fuzzy classifier based on the harmonic mean difference (HMDSOF) to classify the fault feature. The results confirmed the superiority of MPE. Not all the feature information of the rolling bearing vibration signal is sensitive to the tendency of the remaining useful life to decline over time. When using multiscale arrangement entropy as an evaluation feature, the dimensionality of the multiscale permutation entropy value increases as the scale factor increases. There will inevitably be insensitive feature information in the multiscale arrangement entropy value, which affects the accuracy of the remaining useful life prediction of the subsequent rolling bearing. Therefore, the multiscale permutation entropy features extracted from the low-resonance component must be fused to remove the redundant insensitive feature information. To change this situation, new features must be designed to improve the accuracy of the remaining useful life prediction of rolling bearings.

Deep learning has made a qualitative leap in feature learning and fitting capabilities compared with machine learning algorithms in the context of big data. It can be relatively easy to update model parameters in a real time according to the object being tested. Thus, more accurate performance-degradation tracking can be achieved [12]. Based on deep learning, algorithmic models which can predict remaining useful life, such as various neural networks and their extensions, can theoretically be fitted with two layers of neural networks to approximate arbitrary functions. Deep learning techniques such as deep belief networks (DBNs) [13], recurrent neural networks (RNNs) [14], and convolutional neural networks (CNNs) [15] have more powerful representational learning capabilities. They have an ability to learn complex functions that map inputs to outputs directly from raw data without relying entirely on hand-crafted features. Babu et al. [16] proposed a CNN-based method for the RUL prediction of turbofan engines and demonstrated its superiority by comparing it with traditional machine learning methods. Hinchi et al. [17] used CNNs and long short term memory; in the study, CNNs were first used to extract local features from vibration signals, then LSTM networks were used for RUL prediction. Zhang et al. [18] proposed a multiobjective DBN integration and used it to estimate the RUL of turbofan engines. Zhu et al. [19] combined wavelet transforms and CNNs to predict the bearing RUL. Yang Yu et al. [20] put forward a DCNN-based method to localize damages of smart building structures exposed to external harmful excitations. Ince et al. [21] used one-dimensional CNNs for the real-time monitoring of motor faults. With complex and multisource-bearing signals, the convolutional neural network feature-extraction operation cannot fully exploit the feature information of a movement at a single time scale. The problem of information loss may occur in convolutional neural networks during pooling, and this problem will be further aggravated if the feature information extraction is incomplete. Therefore, feature information needs to be extracted at more scales, and should make full use of the multiscale feature information. Li et al. [22] proposed a fault diagnosis method based on the MPE and the multichannel fusion convolutional neural network (MCFCNN). They verified that the technique has high diagnostic accuracy, stability, and speed. Zhang et al. [23] proposed an early fault detection method for rolling bearings based on a multiscale convolutional neural network and a gated circular unit network (MCNN-AGRU), with an attention mechanism which uses a multiscale data-processing method to make the features extracted by CNN more robust. Hou et al. [24] proposed a multiscale convolutional neural network bearing fault diagnosis method based on wavelet transform and a one-dimensional convolutional neural network. Lv D et al. [25] proposed a rolling bearing fault diagnosis method based on a multiscale convolutional neural network (MCNN) and decision fusion. Zhuang et al. [26] proposed a rolling bearing fault diagnosis model based on one-dimensional multiscale deep convolutional neural network. This can broaden and deepen the neural network, enabling it to learn better and have more robust feature representations, while reducing network parameters and the training time. Han et al. [27] proposed a multiscale convolutional neural network (MSCNN) for rolling bearing fault feature extraction. They experimentally demonstrated that MSCNN could learn more robust features than traditional CNN through multiscale convolution operation expressions, reducing the number of parameters and the training time. When feature information is extracted in a convolutional neural network, it is generally fed into the fully connected layer for outputting the final result after simple splicing. This operation weakens the correlation between the features and results in less information for the model to learn. The attention mechanism [28] was proposed by the Google team in 2017 to improve the learning ability of a model when the input sequence is too long. The attention mechanism can attach great importance to the essential features so that the model can focus more on the essential features and improve the model’s learning ability. The attention mechanism can also improve the correlation of multiscale features. The attention mechanism can also explore the correlation of multiscale features, enhance the expression ability of the fused features, and improve the accuracy of the prediction of the remaining useful life of rolling bearings.

In summary, feature extraction is a crucial step in predicting the remaining useful life of rolling bearings. Improving the ability of features to express the declining trend of the remaining useful life of rolling bearings over time is an effective way to improve prediction accuracy. Therefore, resonant sparse decomposition and multiscale permutation entropy methods are used to extract features that can accurately reflect the declining trend of the remaining useful life of rolling bearings. The remaining life prediction model is the main part of the prediction of the residual useful life of rolling bearings; learning the degradation characteristics at a single scale can no longer meet the needs of current rolling bearing residual life prediction demand. Therefore, a multiscale feature learning module was added to the convolutional neural network to enhance the feature learning ability of the model, and the attention mechanism was added to fuse the multiscale degradation feature information, retain the correlation between the degradation feature information in different time scales, and improve the model prediction accuracy.

Feature extraction is the key to predicting the remaining life of rolling bearings. Due to the weak features of early-failure signals, it is challenging to extract sensitive information which reflects the bearings’ decline in performance, which affects the evaluation of the health status of rolling bearings. This method can improve the sensitivity of features to the decline trend of remaining useful life and predict the remaining life of rolling bearings in advance, thus improving the prediction accuracy of the model. It provides an effective technical means for the predictive maintenance of machines.

The main contents are as follows: Section 2 presents a multiscale fusion permutation entropy feature-extraction method; Section 3 presents a MACNN remaining useful life prediction model; Section 4 presents our experimental validation; Section 5 presents our conclusions.

## 2. Multiscale Fusion Permutation Entropy Feature Extraction

The MFPE-based bearing vibration signal feature-extraction method constructs a high-dimensional, entropy-valued feature matrix by calculating the multiscale permutation entropy values of the low-resonance components of rolling bearings. It fully reflects the complexity and instability of the signals from multiple dimensions. The local linear embedding (LLE) algorithm further removes redundant information. The overall method makes up for the imperfect reflection of the characteristics extracted at a single scale on the local trend of rolling bearing life decline and can better improve the prediction accuracy of the remaining useful life of rolling bearings.

### 2.1. Resonance Sparse Decomposition Method

The resonance sparse decomposition method can analyze the resonance properties of a signal. The wavelet basis function library was constructed by an adjustable quality factor wavelet transform approach. The call was sparsely represented by the wavelet basis function library according to the morphological analysis method, and the quality factor, Q, was used as the evaluation method to separate the different components of the signal from each other. When the quality factor, Q, was more extensive, it indicated that the movement bandwidth was narrower, and the movement was in the form of high-resonance periodic vibration. When the quality factor, Q, was smaller, it indicated that the bandwidth of the movement was more expansive, and the movement was in the form of low-resonance transient shock.

The high-resonance element corresponded to the component of continuous oscillation in the movement, that is, the regular vibration movement generated when the bearing ran smoothly. The low-resonance component corresponded to the regular shock component in the movement, that is, the regular shock movement generated when the bearing had regular failures. The low-resonance component can adequately reflect the characteristic information in the movement caused by the fault.

The specific calculation steps of the resonance sparse decomposition algorithm are as follows.

(1) Assume that the input movement is ${X=X}_{1\;}{+X}_{2}$. Set the resonant sparse decomposition parameters quality factor, $Q_{1}\;$ and $Q_{2}$, redundancy factor, $r_{1}\;$ and $r_{2}$, and decomposition level, $J_{1}$ and $J_{2}$, according to the movement characteristics, and construct the wavelet basis function library, $S_{1}$ and $S_{2}$.
(2) Select appropriate weighting coefficients, $\lambda_{1}$ and $\lambda_{2}$, according to the signal-to-noise ratio index so that the different components in the signal can be separated effectively. Set the optimization target as shown in Equation (1).
  (1) $${J(\omega }_{1}{,\omega }_{2})=\underset{\omega_{1}{,\omega }_{2}}{\mathrm{argmin}}\left\{\|X-S_{1}\omega_{1}-S_{2}\omega_{2}\|{+\lambda }_{1}\|\omega_{1}\|{}_{1}{+\lambda }_{2}\|\omega_{2}\|{}_{1}\right\}$$
  where $\omega_{1}$ and $\omega_{2}$ are the matching coefficients of wavelet bases $S_{1}$ and $S_{2}$.
(3) The best-matching coefficients $\omega_{1}^{*}$ and $\omega_{2}^{*}$ are obtained by solving the optimization problem of Equation (1), and the high-resonance component $X_{1}$ and the low-resonance component $X_{2}$ are obtained by combining the best-matching coefficients $\omega_{1}^{*}$ and $\omega_{2}^{*}$ with the wavelet basis function library for calculation.

### 2.2. Multiscale Permutation Entropy

**Figure 1:**
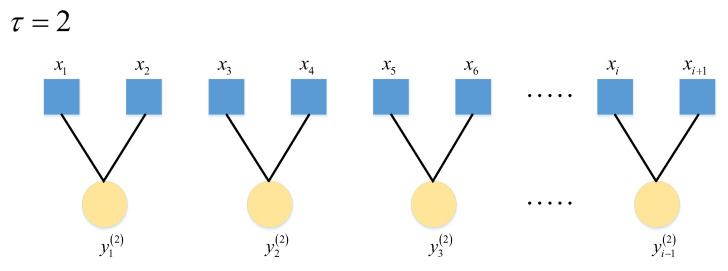
Schematic diagram of coarse granulation.

Multiscale permutation entropy avoids the limitation of the permutation entropy to evaluate the information of temporal characteristics of signals from a single scale by coarsening the input signal, τ=2. The coarse granulation treatment at the time is shown in Figure 1.

The specific calculation steps for multiscale permutation entropy are as follows.

(1) Suppose the input signal sequence is $X_{N}=\left\{x_{1}{,x}_{2},\cdots {,x}_{N}\right\}$ Coarse granulation is shown in Equation (2).
  (2) $$y_{j}^{(\tau )\;}=\frac{1}{\tau }\overset{j\tau }{\underset{i=(j-1)\tau +1}{\sum }}x_{i},1\;\leq \;j\;\leq \;\frac{N}{\tau }.\;$$
  where τ is the scale factor; $y_{j}^{\left(\tau \right)}$ is the coarse-graining sequence.
(2) The coarse-grained sequence $y^{\left(\tau \right)}$ phase space is reconstructed to obtain the multiscale sequence, as shown in Equation (3).
  (3) $$Y_{i}^{\left(\tau \right)}=\left\{y_{i+1}^{\left(\tau \right)}{,y}_{i+s}^{\left(\tau \right)},\cdots {,y}_{i+(m-1)s}^{\left(\tau \right)}\right\}$$
  where $Y_{i}^{\left(\tau \right)}$ is the multiscale sequence; m is the embedding dimension; s is the time delay sparsity.
(3) Arrange the multiscale time series $Y_{i}^{\left(\tau \right)}$ in ascending order and record the index $\theta_{j\;}=\left\{j_{1}{,j}_{2},\cdots {,j}_{m}\right\}$ of each short time series after the ascending order. There are m! permutations of each short time series. Count the number of occurrences of each permutation $N_{l}$ and calculate the frequency of each permutation, as shown in Equation (4).
  (4) $$P_{l}^{\left(\tau \right)}=\frac{N_{l}}{N/\tau -m+1}$$
(4) The multiscale permutation entropy is obtained by calculating the permutation entropy of the multiscale time series, as shown in Equation (5) [29].
  (5) $${\mathrm{MPE}}^{\left(\tau \right)}=-\overset{m!}{\underset{l=1}{\sum }}P_{l}^{\left(\tau \right)}{\mathrm{lnP}}_{l}^{\left(\tau \right)}$$

### 2.3. Multiscale Fusion Permutation Entropy

The multiscale permutation entropy reconstructs the movement by coarse granulation and phase space reconstruction operations. It can obtain the feature information of the movement on different time scales. The problem of incomplete feature information on a single dimension was improved. It can improve the accuracy of the remaining useful life prediction. Due to the use of the sliding window slicing processing method to construct the short time series matrix, a partial overlap of the movement was caused. Although this operation can enrich the feature information in the signal, it can also cause the redundancy of features in the signals that are insensitive to the decline trend of the remaining useful life of the rolling bearing. In turn, this causes feature redundancy in the high-dimensional, multiscale permutation entropy feature matrix. Therefore, dimensionality reduction is needed to retain the primary feature information in the high-dimensional feature matrix.

The specific steps of the multiscale fusion permutation entropy feature-extraction method are shown below:

(1) The input data are known to comprise a multiscale permutation entropy matrix $\mathrm{MPEM}=\left[E_{1}{,E}_{2},\cdots {,E}_{z}\right]$, which contains z-dimensional multiscale permutation entropy vectors, and the objective is to reduce the multiscale permutation entropy matrix to d dimensions. The k nearest neighbors of an entropy value $e_{i}$($e_{i}\;\in \;E_{i}$) are found according to the Euclidean distance. The linear relationship between the entropy value $e_{i}$ and the k nearest neighbors are established after the k nearest neighbors are found. The loss function is shown in Equation (6).
  (6) $$J\left(\omega \right)=\overset{m}{\underset{i=1}{\sum }}\|e_{i}-\underset{j\in K\left(i\right)}{\sum }\omega_{\mathrm{ij}}e_{j}{\|}_{2}^{2}$$
  where $K\left(i\right)$ denotes the k nearest neighbor samples with an entropy value $e_{i}$; $\omega_{\mathrm{ij}}$ is the linear weight coefficient, which is generally normalized to satisfy the condition shown in Equation (7). For the entropy value of the k nearest neighbor samples that are not in the entropy value $e_{i}$, the weight coefficient will be made to be 0, and the weight coefficient will be extended to the dimensionality of the whole dataset.
  (7) $$\underset{j\in K\left(i\right)}{\sum }\omega_{\mathrm{ij}\;}=1$$
(2) Calculate the covariance matrix $Z_{i}$ in the space of k nearest neighbor samples, as shown in Equation (8), and find the corresponding vector of weight coefficients $W_{i}$, as shown in Equation (9).
  (8) $$Z_{i\;}=\left(x_{i}-x_{j}\right){\left(x_{i}-x_{j}\right)}^{T}$$
  (9) $$W_{i}=\frac{Z_{i}^{-1}1_{k}}{1_{k}^{t}Z_{i}^{-1}1_{k}}$$
  where $1_{k}$ is a vector with the k-dimensional value of 1.
(3) The weight coefficient vector W is constructed as the weight coefficient matrix $W_{i}$, from which the conditioned matrix M is calculated as shown in Equation (10).
  (10) $$M=\left(I-W\right){\left(I-W\right)}^{T}$$
  where I is the constraint that ensures that the entropy value retains the original feature information as much as possible after dimensionality reduction. $I=\frac{1}{s}\sum_{i=1}^{s}y_{i}y_{i}^{T}$; $y_{i}$ is the fusion entropy value obtained after dimensionality reduction.
(4) Compute the first d+1 eigenvalues of the conditional matrix M and compute the eigenvector $\left\{y_{1}{,y}_{2},\cdots {,y}_{d+1}\right\}$ corresponding to these d+1 eigenvalues.
(5) The matrix consisting of the second eigenvector $y_{2}$ to the d+1st eigenvector is the multiscale fusion permutation entropy matrix, $\mathrm{MFPE}=\left\{y_{2}{,y}_{3},\cdots {,y}_{d+1}\right\}$, obtained by dimensionality reduction.

## 3. MACNN Remaining Useful Life Prediction Model

The MACNN remaining useful life prediction model consists of a multiscale convolutional learning module and a remaining useful life forecast module. In the MACNN model, the multiscale fusion permutation entropy feature matrix was used as the input. The detailed data were automatically learned and detected by constructing the multiscale convolutional learning module. The primary information for determining the remaining useful life was fused and highlighted by a self-attentive mechanism and input into the module for remaining useful life prediction.

### 3.1. Multiscale Convolution Module

**Figure 2:**
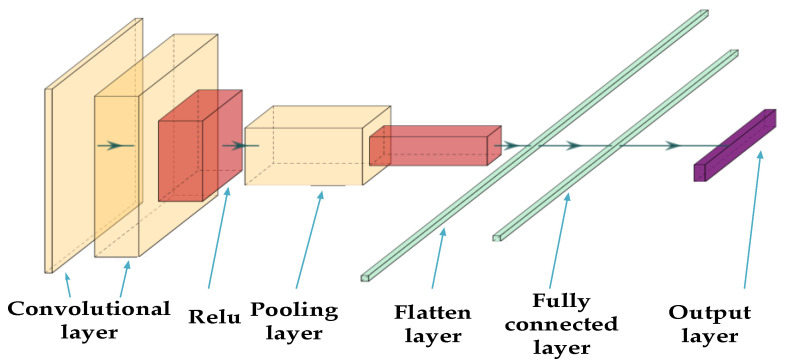
Convolutional neural network structure.

A convolutional neural network is a feed-forward neural network, and the main structure of a convolutional neural network is shown in Figure 2.

**Figure 3:**
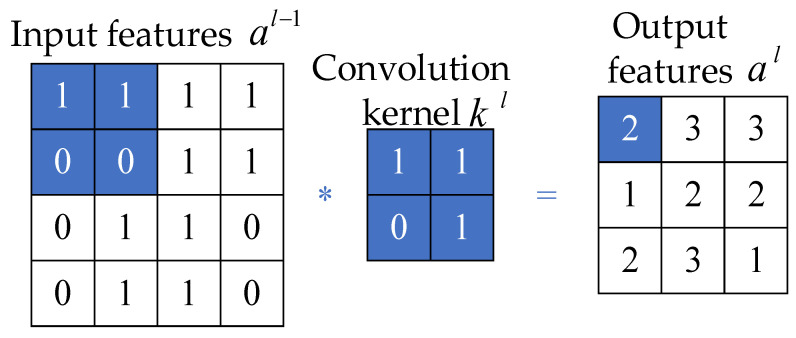
Convolution calculation schematic.

(1) Convolutional layer:

Through feature extraction in the convolutional layer, a convolutional neural network can capture the deep features of interconnections between the input data. In the conventional layer, multiple convolution kernels are passed that are updated with model training. The output feature matrix of the convolution layer is obtained by performing dot product operations between convolution kernels and corresponding elements of the feature matrix covered by convolution kernels. Each output feature matrix is calculated from multiple input feature matrices of the previous convolutional layer. The output value $a_{j}^{l}$ of the j-th cell of the convolution layer l is shown in Equation (11), and the convolution calculation is shown in Figure 3 [30].

(11) $$a_{j}^{l}=f\left(b_{j}^{l}+\underset{i\in M_{j}^{l}}{\sum }a_{j}^{l-1}{*k}_{\mathrm{ij}}^{l}\right)$$

where $b_{j}^{l}$ is the bias, k is the convolution kernel, and the parameters are updated when feedback updates are performed after each round of model training.

There are two problems in the convolution calculation process.

(1) The output feature matrix size declines after each convolution computation compared with the input feature matrix. When the input feature matrix has a small size, or multiple consecutive convolution calculations are executed, the amount of information in the output feature matrix will be minimal, resulting in the loss of useful information and altering the reliability of subsequent tasks.
(2) Edge features of the input feature matrix. The number of calculations is less, which means that the edge information in the input feature matrix will be less involved in the analysis of the final output result. It causes the edge information of input features to be lost.

**Figure 4:**
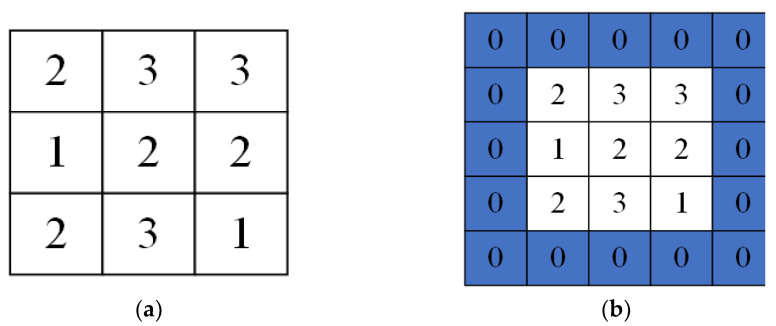
(**a**) Valid padding and (**b**) same padding.

To solve these two problems, the input feature matrix is usually padded, and the main padding operations are valid padding and same padding. Valid padding is used directly to convolve the image with the convolution kernel of the input feature matrix. It is used when the input feature matrix size is significant and needs to be reduced. The same padding is used to restore the original size of the output feature matrix by padding 0. The output feature matrix, after filling with valid and same padding, is shown in Figure 4.

**Figure 5:**
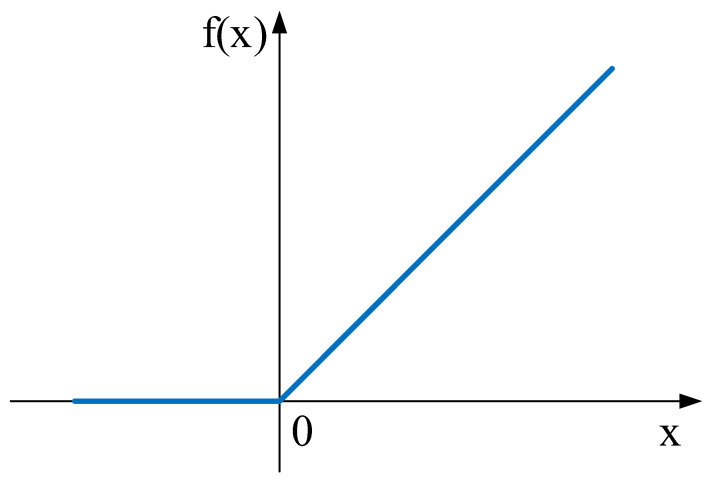
ReLU activation function image.

(2) ReLU layer:

It is essential to add an activation function after the convolutional layer to enhance the nonlinear expression ability of the input movement and make the learned features more distinguishable. In recent years, rectified linear unit (ReLU), which is the most widely used activation unit, has been applied to CNNs to accelerate the convergence. Combined with the backpropagation learning method to adjust parameters, the ReLU makes shallow weights more trainable [31]. The ReLU function is calculated as shown in Equation (12), and the function image is shown in Figure 5.

(12) $$f\left(x\right)=\max\left(0,x\right)$$

The ReLU activation function has the following advantages:

(1) Smaller computation: Because the ReLU function does not involve complex operations, it can save a lot of computation time and can improve the efficiency of the overall network model.
(2) Prevent gradient decay: When the result of the activation function is small, training parameters are updated to a lesser extent or are not updated. In contrast, the ReLU function has a result of 1 in the activation function interval, avoiding this phenomenon.
(3) The overfitting phenomenon is mitigated, as shown in Figure 5. When the feature value obtained after the calculation is less than zero, the ReLU activation function will be assigned to zero. Although this may cause information loss, it also increases the sparsity of the model, reduces the learning ability of the model, and enhances the generalization ability of the model.

The ReLU activation function performs poorly for data with more negative values in input features. In the continuing life forecast for rolling bearings, the input data used are all positive, and output target values are all greater than, or equal to, zero. Consequently, if initialization weight parameters of the control model are more significant than zero, the shortcomings of the ReLU activation function can be prevented, and the computational efficiency and accuracy of the model can be improved.

**Figure 6:**
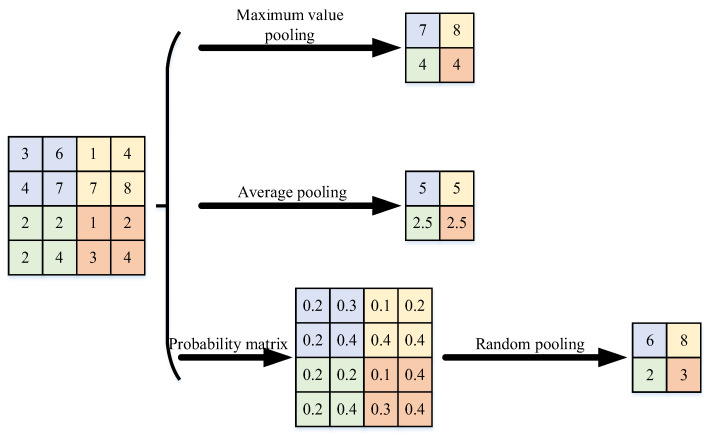
Different pooling function calculation methods.

(3) Pooling layer:

The pooling layer and the convolutional layer form the feature-extraction module. The pooling layer can reduce the redundancy of the feature matrix and alleviate the overfitting phenomenon. The activation value $a_{j}^{l}$ in pooling layer l is calculated as shown in Equation (13).

(13) $$a_{j}^{l}=f\left(b_{j}^{l}{+\beta }_{j}^{l}{\mathrm{down}(a}_{j}^{l-1}{,M}^{l})\right)$$

where $b_{j}^{l}$ is the bias; $\beta_{j}^{l}$ is the multiplicative remaining useful; $M^{l}$ is the pooling window size; down() denotes the pooling function; the commonly used pooling function is calculated as shown in Figure 6.

(4) Flatten layer:

The flatten layer converts the feature matrix output from the feature-extraction module into a one-dimensional feature vector so that the features meet the input dimension requirements of the subsequent, fully connected layers.

**Figure 7:**
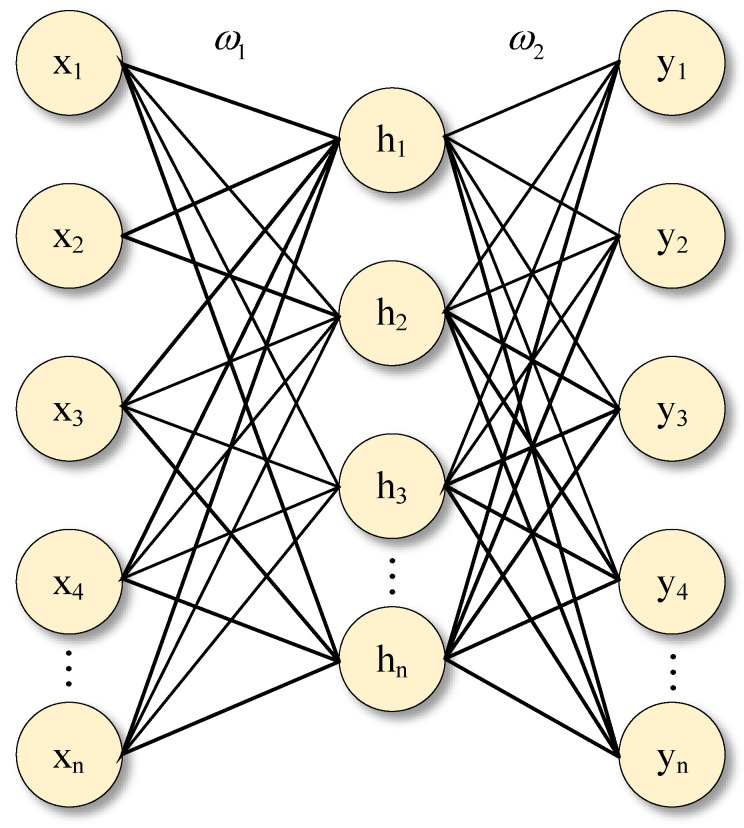
The fully connected layer structure.

(5) Fully connected layer:

In a convolutional neural network, after feature-extraction operations such as convolution and pooling, the output feature matrix is converted into a one-dimensional feature vector by the flatten layer, which is input to the fully connected layer for classification or prediction tasks. The fully connected layer in a convolutional neural network is the same as a multilayer perceptron. The fully connected layer discovers the local information contained in features. The structure of the fully connected layer is shown in Figure 7 and is calculated as shown in Equation (14).

(14) $$\begin{array}{c} h_{n}{=\omega }_{1}x_{n}{+b}_{n}\\ y_{n}=f\left(\omega_{2}h_{n}{+b}_{n}\right) \end{array}$$

where ω is the weight between each hidden layer, b is the bias, and f() is the activation function.

**Figure 8:**
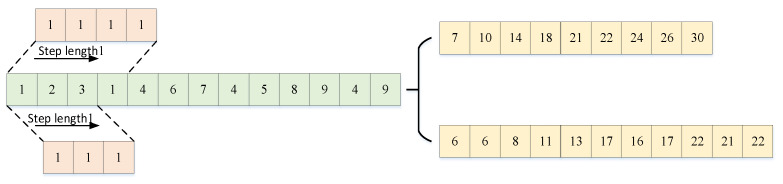
The one-dimensional convolutional neural network convolution process.

In the task of predicting the continuing life of bearings, the input is a one-dimensional feature vector. So, a one-dimensional convolutional neural network model is used as the base model for remaining useful life prediction. The one-dimensional convolutional neural network convolution process is illustrated in Figure 8. Convolutional kernels of dimensions (1, 4) and (1, 3) are used to convolve the input sequence under the condition of the concurrent length of the same value, respectively. The input sequence is an ascending sequence with fluctuations in the middle. From the convolution results, feature sequences calculated by convolution kernels of different scales reflect the feature trends of the input sequence differently. The feature sequence extracted from the convolution kernel of size (1, 4) reflects the increasing trend of the input sequence well but does not reflect the fluctuation trend in the input sequence. The feature sequence extracted from the convolutional kernel of size (1, 3) reflects the rising and fluctuating trends of the input features but does not reflect either direction significantly.

**Figure 9:**
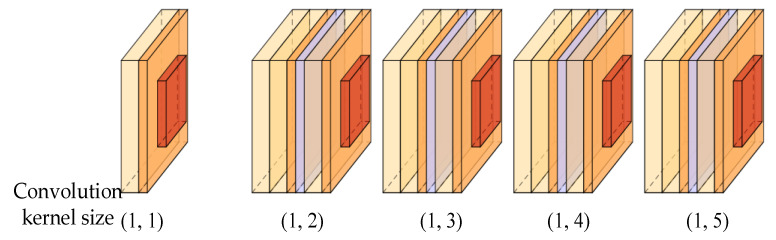
Schematic diagram of the multiscale feature-extraction module.

Convolutional neural networks often do not reflect the feature information well when the feature extraction is performed on input features at a single scale. Therefore, a multiscale convolutional module is proposed for feature learning, which consists of four conventional modules with different convolution kernel sizes in parallel. Each convolutional module consists of three layers, two ReLU activation layers, one BN layer, and one pooling layer [32], as shown in Figure 9. With the multiscale convolution module, the resolution of the features can be improved, which improves the remaining useful life prediction accuracy.

Suppose $x^{l-1}\;\in {\;R}^{H\times 1\times C}$ and $k^{l}\;\in \;R^{F\times 1\times C\times N}$ denote the input vector and the learnable convolutional kernel, respectively, where H denotes the input vector length, C denotes the number of input channels, 1×F represents the size of the convolutional kernel, and 1×F represents the number of convolutional kernels. Then, the n-th feature vector of the l-th convolutional layer is shown in Equations (15) and (16).

(15) $$x_{n\;}^{l}=\sigma \left(u_{n}^{l}\right)$$

(16) $$u_{n}^{l}{=k}_{n}^{l}{*x}^{l-1}{+b}_{n}^{l}=\overset{C}{\underset{c=1}{\sum }}k_{n,c}^{l}{*x}_{c}^{l-1}{+b}_{n}^{l}$$

where $\sigma \left(\cdot \right)$ denotes the Relu activation function, $u_{n}^{l}$ denotes the output of the convolutional layer, * denotes the convolutional computation, $k_{n}^{l}$ denotes the n-th convolutional kernel of the l-th convolutional layer, and $b_{n}^{l}$ denotes the bias.

In the multiscale convolution module, the pooling layer is set after the third convolution layer. The main feature information learned is obtained by the maximum pooling operation after passing through the convolution layer, as shown in Equation (17).

(17) $$y_{n}^{l}=\mathrm{Maxpooling}\left(y_{n}^{l-1},p,s\right)$$

where $y_{n}^{l-1}$ is the output of the n-th feature map, $\mathrm{Maxpooling}\left(\cdot \right)$ denotes the maximum pooling function, p denotes the pooling layer size, and s denotes the number of steps.

### 3.2. Attentional Mechanisms

**Figure 10:**
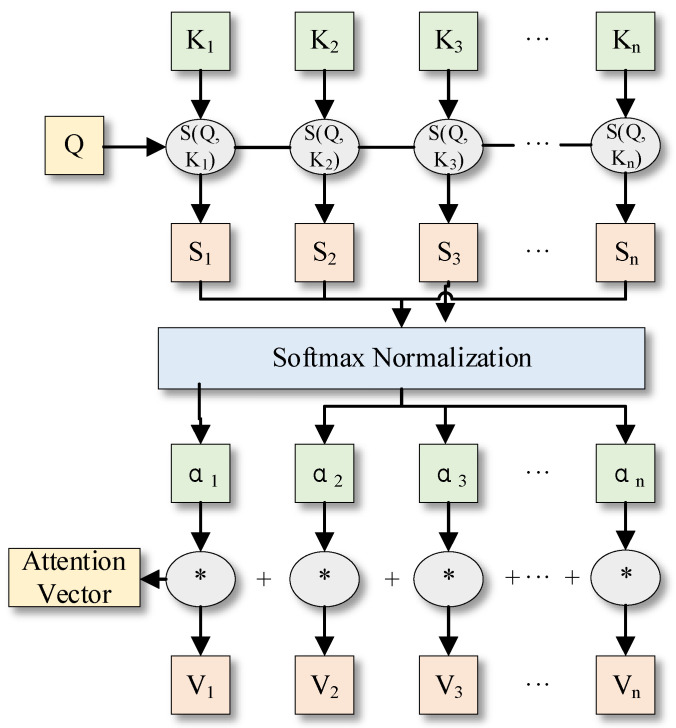
Diagram of attention mechanism.

The attention mechanism is an algorithm inspired by the human visual attention mechanism, which assigns different attention weights to each feature, thus allowing the model to focus more on more critical features, as shown in Figure 10.

The commonly used weight calculation methods in the attention mechanism are additive, dot product, and scaled dot product bilinear calculations, as shown in Equation (18).

(18) $${\mathrm{Point}\;\mathrm{product}\;\mathrm{calculation}\;s(K}_{i}{,Q)=k}_{i}{}^{T}q$$

where Q is the state of the last time step when the model performs time series prediction. K is the state of each time step when the model performs time series prediction. $s(K_{i},Q)$ is the attention weight calculation mechanism that calculates the correlation between Q and K. d is the dimensionality of the data in the time step. $\alpha_{i}$ is the estimated attention weight, which suggests the importance of the time step to the overall time series feature expression importance; V is the same as K, which suggests the state of each time step when the model performs time series prediction.

### 3.3. Remaining Useful Life Prediction Module

**Figure 11:**
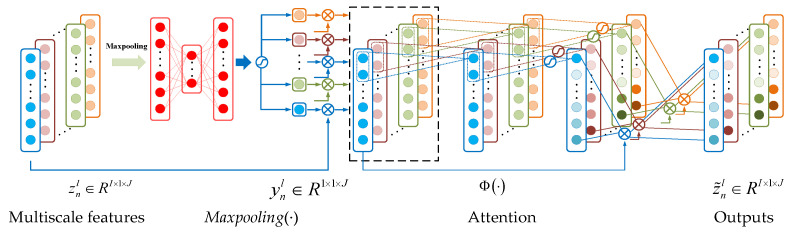
Schematic diagram of the remaining useful life prediction module.

The remaining useful life prediction module consists of the attention module and the fully connected neural network. The attention module is constructed to effectively fuse the feature information learned by the multiscale convolutional module and highlight the part of it that is relevant to the remaining useful life. As shown in Figure 11, features extracted by the multiscale convolution module are used as the input of the remaining useful life prediction module, assuming that $z_{n}^{l}\;\in {\;R}^{I\times 1\times J}$ denotes the input feature vector; $\alpha^{l}\;\in {\;R}^{I\times 1\times J}$ indicates the attention weight, where I is the length of the feature vector; J=N × C indicates the number of feature vectors. The attention module features are fused, as shown in Equation (19).

(19) $${\tilde{z}}^{l}{=\alpha }^{l}⊗z^{l-1}=\Phi \left(z^{l-1}\right)⊗z^{l-1}$$

where ⊗ denotes the corresponding element multiplication operation in the matrix, ${\tilde{z}}^{l}\in R^{I\times 1\times J}$ is the fused eigenvector, ϕ(·) indicates the attention weight calculation function, and the scaled dot product calculation function is used in this paper.

Finally, the fused feature vectors are input to the fully connected neural network for remaining useful life prediction. The fully connected neural network in this paper contains two hidden layers containing 64 and 128 nodes, respectively. The fully connected neural network prediction is calculated in Equation (20).

(20) $$\begin{array}{c} h_{m}^{n}{=\omega }_{n}^{n}{\tilde{z}}^{l}{+b}_{m}\\ y_{p}{=f(\omega }_{n}^{n}h_{m}^{n}{+b}_{m}) \end{array}$$

where $\omega_{m}^{n}$ denotes the weight of the n-th node of the m-th hidden layer, $h_{m}^{n}$ is the output of the n-th node of the m-th hidden layer, $f\left(\cdot \right)$ represents the activation function after the hidden layer, and $y_{p}$ is the final predicted output.

### 3.4. Model Parameters and Structure

**Figure 12:**
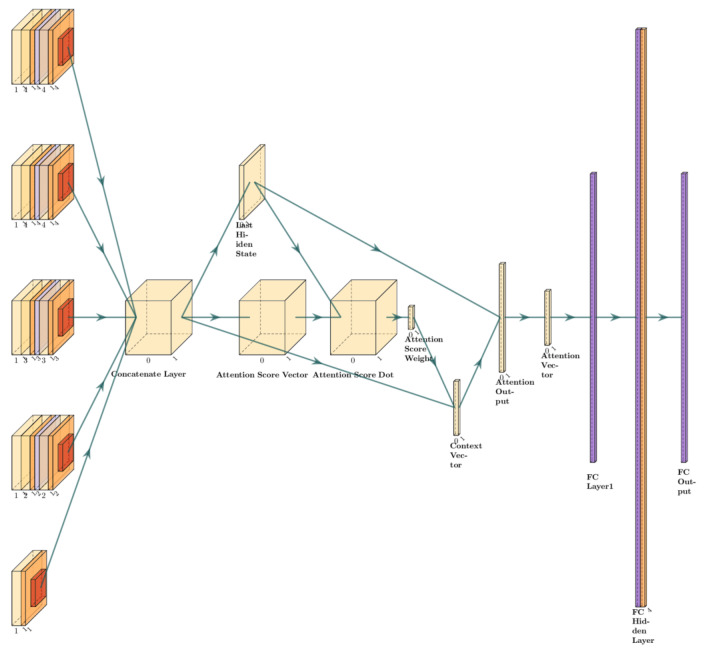
MACNN model structure diagram.

The MACNN remaining useful life prediction model of rolling bearings is built on a multiscale feature-extraction module with an attention mechanism. The overall model first uses a convolution kernel of size (1, 1) to extract the shallow features of the input data. Then, four convolution modules are used to remove the deep features at different scales, respectively. Because of the large number of parameters in the overall model, to prevent the model from overfitting, the remaining join is used to stitch the shallow features with the extracted deep features. Spliced multiscale features are input into the attention fusion layer to obtain the fused attention feature vector, which is input to the fully connected layer to obtain the final prediction results. The specific parameters of the overall model are shown in Table 1, and the model structure is shown in Figure 12.

### 3.5. Overall Methodology Flow

**Figure 13:**
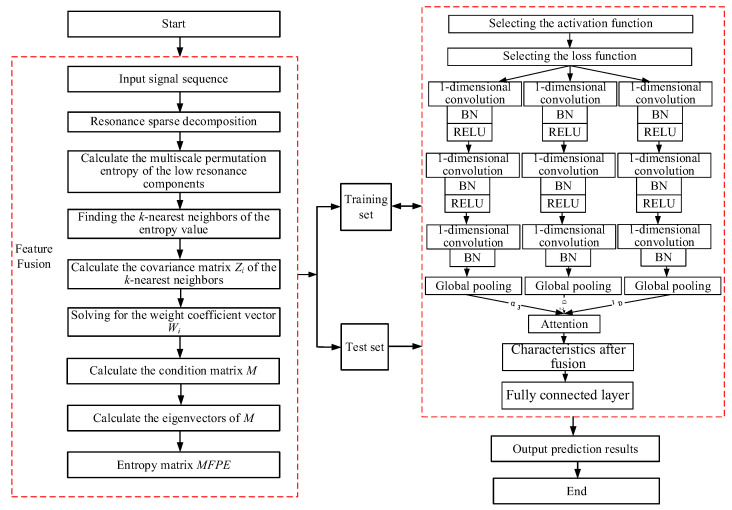
Overall method flow chart.

The rolling bearing remaining useful life prediction model using the MFPE–MACNN adequately reflects the complexity and instability of the movement from multiple dimensions. The overall method makes up for a defect: the features extracted at a single scale do not fully reflect the local trend of decline of the life of rolling bearings. It can improve the accuracy of the remaining useful life prediction for rolling bearings. Based on the construction of the multiscale fusion permutation entropy with low-resonance components as features for assessing the bearing life decline trend, the multiscale feature-extraction module and the attention mechanism are added to the one-dimensional convolutional neural network to enhance the learning ability of the model for multiscale features. A multiscale attentional convolutional neural network rolling bearing remaining useful life prediction model is built. The overall method flow chart is shown in Figure 13, and the specific steps are as follows.

Step 1: The resonant sparse decomposition of the input movement sequence as $X_{N}=\left\{x_{1}{,x}_{2},\cdots {,x}_{N}\right\}$ yields the high-resonance component and the low-resonance component.

Step 2: The short time series multiscale permutation entropy values are calculated to the entropy matrix $\mathrm{MPEM}=\left[E_{1}{,E}_{2},\cdots {,E}_{z}\right]$ for the low-resonance components.

Step 3: Find the nearest neighbor with entropy value k and calculate the covariance matrix $Z_{i}$ and the corresponding weight coefficient vector $W_{i}$ in the sample space of the k nearest neighbors.

Step 4: Construct the weight coefficient vector $W_{i}$ into the weight coefficient matrix W, and use it to calculate the conditioned matrix M.

Step 5: Calculate the first d+1 eigenvalues of the conditioned matrix M and calculate the eigenvector $\left\{y_{1}{,y}_{2},\cdots {,y}_{d+1}\right\}$ corresponding to these d+1 eigenvalues.

Step 6: The matrix consisting of the second eigenvector $y_{2}$ to the d+1st eigenvector is the multiscale fusion permutation entropy matrix $\mathrm{MFPE}=\left\{y_{2}{,y}_{3},\cdots {,y}_{d+1}\right\}$ obtained by dimensionality reduction.

Step 7: Determine the size of multiple convolutional kernels, select the loss function, select the activation function, and determine the number of layers of the multiscale convolutional kernel for the remaining useful life prediction model of the multiscale convolutional neural network.

Step 8: Incorporate the attention mechanism into the remaining useful life prediction model of the multiscale convolutional neural network to form the remaining useful life prediction model of the multiscale convolutional attention neural network.

Step 9: The extracted feature matrix $\mathrm{MFPE}=\left\{y_{2}{,y}_{3},\cdots {,y}_{d+1}\right\}$ of the training set is input to the remaining useful life prediction model of the multiscale convolutional attention neural network to obtain the output error, and the error is backpropagated to update the prediction model parameters.

Step 10: After the parameters of the prediction model are updated to reach the optimal requirements, the test set is input to the MACNN prediction model to complete the prediction of the remaining useful life of the rolling bearing.

## 4. Experiments

### 4.1. Simulation Experiment Validation

To verify the effectiveness of the proposed feature enhancement and feature-extraction method, a feature extraction simulation experiment was set up. The simulation movement was composed of the superimposed shock movement and the modulated movement. The sub-constructions of the shock movement and the modulated movement are shown in Equations (21) [33] and (22). To simulate the failure of the bearing under operating conditions in order to find the best method for extracting the bearing vibration signal features, the sampling frequency was set to 8192 Hz, and the number of sampling points was set to 4096.

(21) $${y=y}_{0}e^{-{2\pi \mathrm{gf}}_{n}t_{0}}\sin\left({\pi f}_{n}\sqrt{{(1-g}^{2})}{(t}_{0}-\mathrm{KT})\right)$$

(22) $$x=\left(1+\cos\left({2\pi f}_{r}t\right)\right)\cos\left({2\pi f}_{z}t\right)$$

where $y_{0}$ is the displacement constant, set to 5; g is the damping coefficient, set to 0.5; $f_{n}$ is the intrinsic frequency, set to 1000 Hz; $t_{0}$ is the single-cycle sampling interval; K is the number of repetitions of the shock movement; T is the repetition period, set to 0.025 s; $f_{r}$ is the amplitude modulation frequency, set to 70 Hz; $f_{z}$ is the carrier frequency, set to 560 Hz.

**Figure 14:**
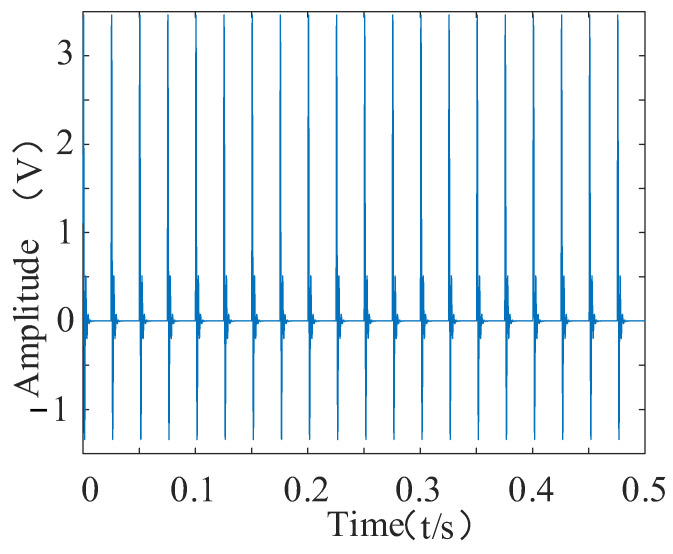
Time domain diagram of the shock.

**Figure 15:**
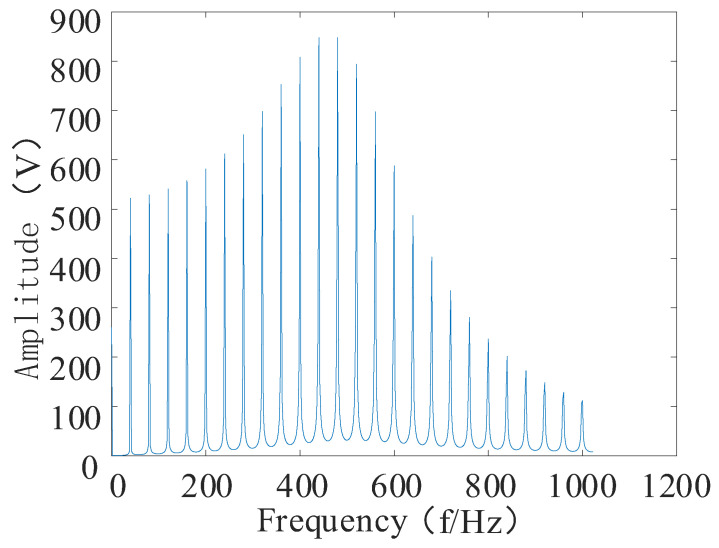
Shock signal spectrogram.

The time and frequency domain diagrams of the shock movements are shown in Figure 14 and Figure 15, respectively.

**Figure 16:**
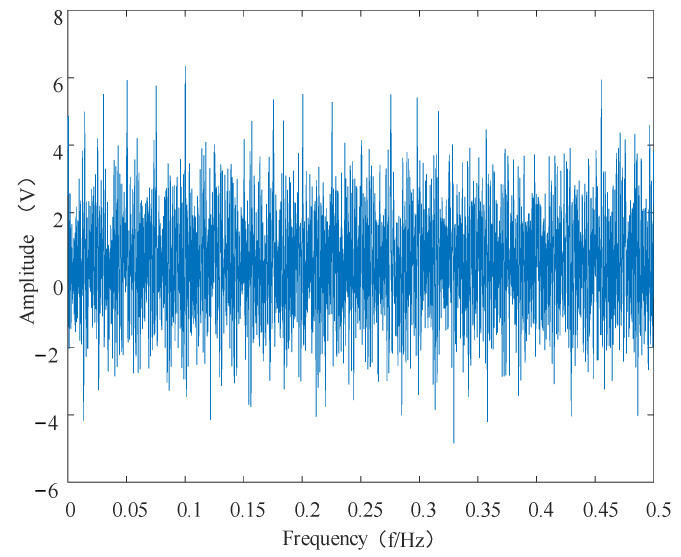
Time domain diagram of the simulated signal.

**Figure 17:**
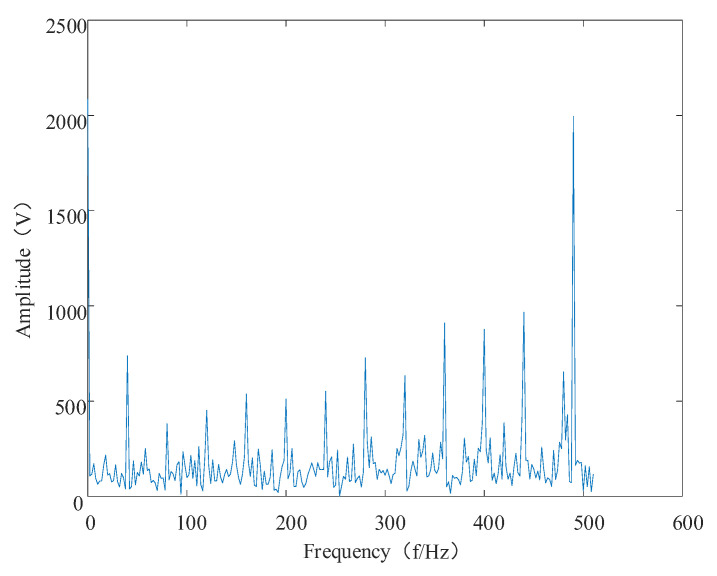
Simulated signal envelope spectrum.

The time domain diagram and envelope spectrum of the synthesized original data are shown in Figure 16 and Figure 17.

**Figure 18:**
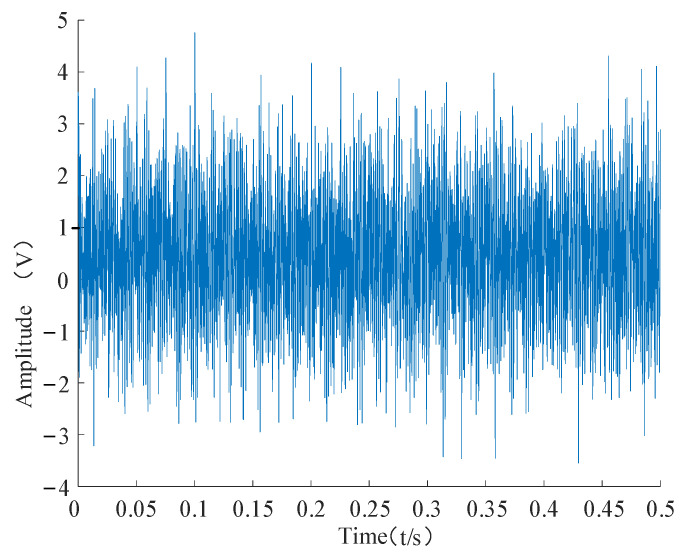
High-resonance component of the simulated signal.

**Figure 19:**
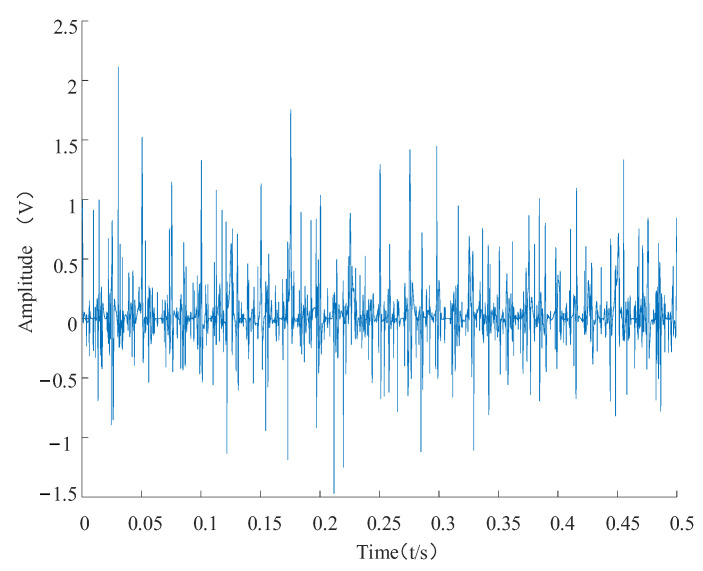
Low-resonance component of the simulated signal.

The sparse decomposition high-resonance quality factor, $Q_{1}$, was set to 3; redundancy, $r_{1}$, was set to 3; the number of decomposition layers, $J_{1}$, was set to 27; the low-resonance quality factor, $Q_{2}$, and the redundancy, $r_{2}$, were set to 3; the number of decomposition layers, $J_{2}$, was set to 7 [34]. The high-resonance component retrieved after decomposition and the moderate-resonance components are depicted in Figure 18 and Figure 19, respectively. As shown in Figure 18, the high-resonance component is mainly the periodic oscillation component in the simulated signal. As shown in Figure 19, the low-resonance component principally contains the shock features in the simulated signal.

**Figure 20:**
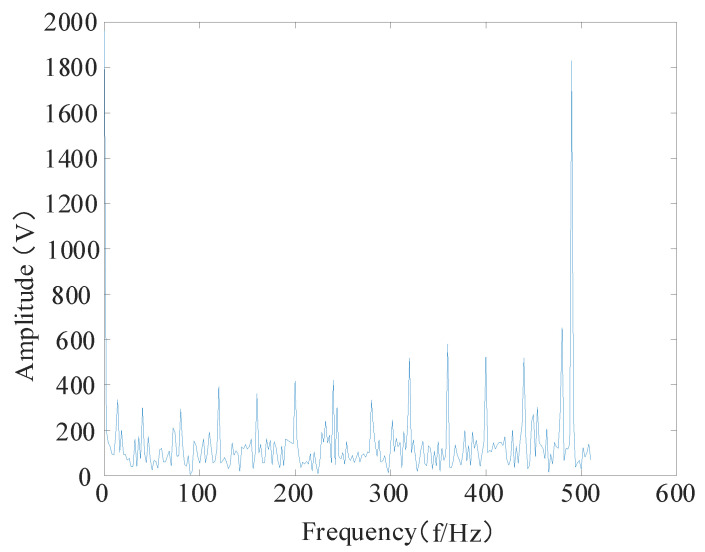
High-resonance component envelope spectrum.

**Figure 21:**
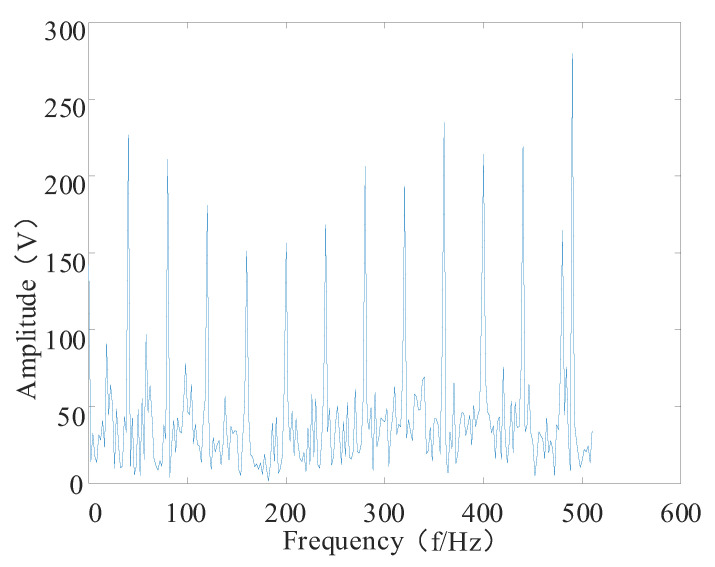
Low-resonance component envelope spectrum.

The envelope spectra of the high-resonance component and the low-resonance component are analyzed as shown in Figure 20. The overall trend of the envelope spectrum of the high-resonance component is no different compared to the simulated signal envelope spectrum, except for a slight decrease in amplitude. The overall amplitude of the shock component in the low-frequency band is not prominent, and features reflecting the decline in the remaining useful life of the bearing cannot be better extracted in the subsequent feature extraction. As shown in Figure 21, the overall amplitude of the low-resonance component envelope spectrum decreases compared with the broad simulated signal envelope spectrum. However, the fault shock component in the low-frequency band of the envelope spectrum is more evident in the low-frequency band.

**Figure 22:**
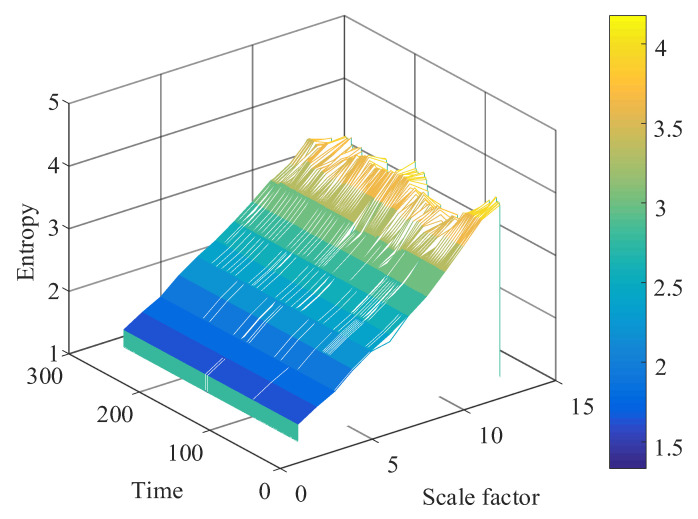
Simulated signal multiscale permutation entropy matrix.

The multiscale permutation entropy matrix of signals with low-resonance components was calculated, and the obtained multiscale permutation entropy matrix is shown in Figure 22.

**Figure 23:**
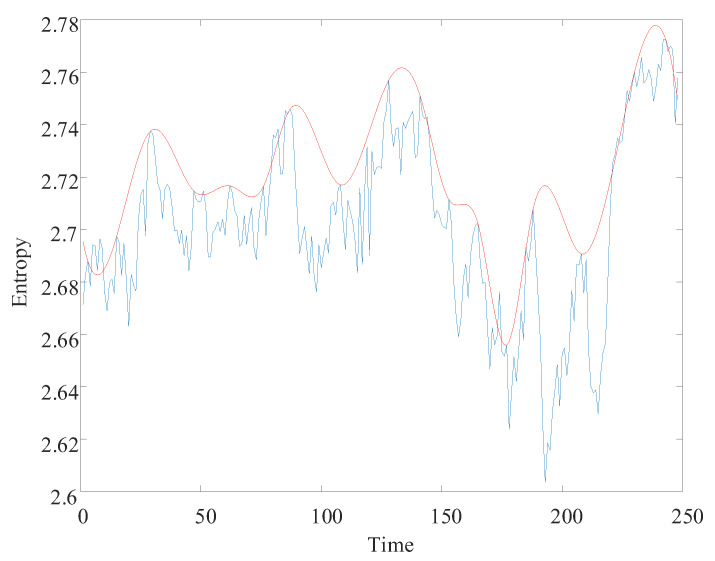
Simulated signal multiscale fusion permutation entropy curve.

Using the locally linear embedding algorithm, the high-dimensional multiscale alignment entropy value matrix is downscaled to a one-dimensional vector. The multiscale fused permutation entropy feature vector reflecting the remaining useful life decline trend is obtained, as shown in Figure 23. The red curve facilitates the observation of the multiscale fusion permutation entropy trend feature curve. The envelope of the multiscale fusion alignment entropy characteristic curve is drawn. With the occurrence of bearing failure, the remaining useful life of the bearing declines over time. The overall multiscale fusion permutation entropy value shows an increasing trend and negatively correlates with the remaining useful life. Due to the more intensive frequency of the impact fault signal in the simulation signal, the multiscale fusion alignment entropy value fluctuates more.

### 4.2. Cincinnati Data Validation

**Figure 24:**
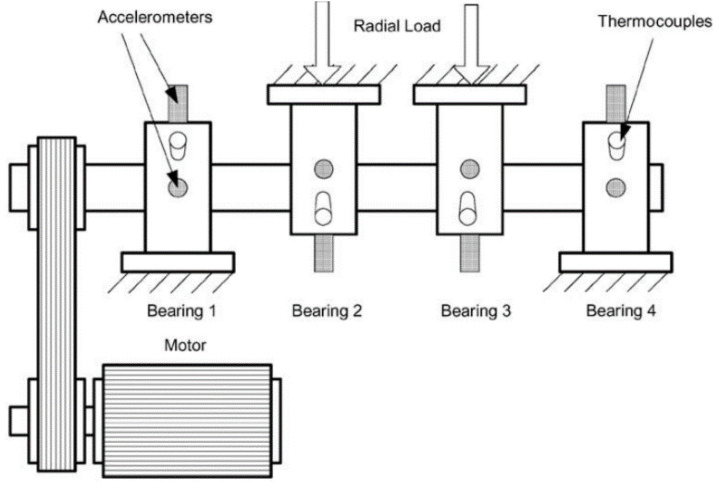
Bearing test rig and sensor placement illustration.

To verify the proposed method effectiveness, the Cincinnati open data were used as experimental data [35]. Features were extracted as input using the multiscale fusion alignment entropy feature-extraction method. Then, degradation features among them were removed by the depth of the convolution module in each scale of the model. Bearing degradation features were received by the attention fusion. Finally, the life prediction results were received by the fully connected layer. The test stand was fitted with four Rexford ZA-2115 bearings, each with 16 rollers in the raceway. The roller diameter was 0.331 cm, the pitch diameter was 2.815 cm, the contact angle was 15.17°, the speed was 2000 r/min, and the radial load was 26.66 KN. One acceleration sensor was installed on the axial and radial direction of each bearing, and the sampling frequency was 20 kHz, as shown in Figure 24.

According to characteristics of the input data, the parameters of this experiment were set as shown in Table 2.

**Figure 25:**
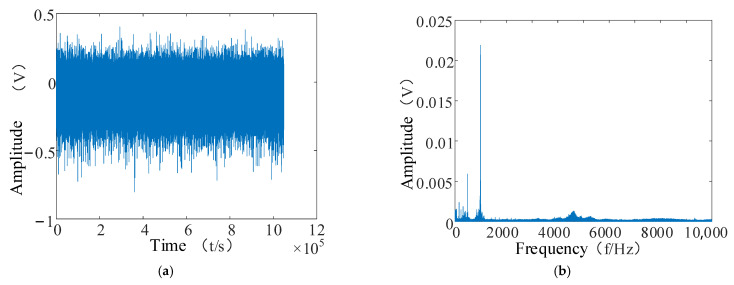
No. 3 bearing time and frequency diagram. (**a**) Time domain diagram of bearing No. 3; (**b**) bearing No. 3 spectrogram.

Only the No. 3 and No. 4 bearings were destroyed in the first experimental data stage in Cincinnati: the No. 3 bearing was destroyed in the inner ring, and the No. 4 bearing was destroyed in the rolling element. Therefore, in this paper, the No. 3 bearing was chosen to analyze the time domain and frequency domain plots to verify the effectiveness of the proposed RUL prediction method. The time domain and spectrum plots are shown in Figure 25.

**Figure 26:**
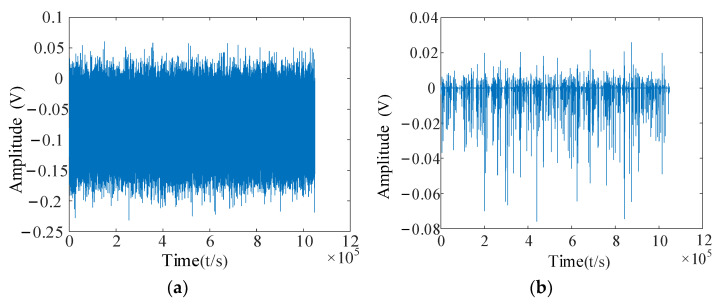
Resonant sparse decomposition results for bearing No. 3. (**a**) No. 3 bearing high-resonance fraction; (**b**) No. 3 bearing low-resonance component.

The resonance showed sparse decomposition after preprocessing the vibration data of the No. 3 bearing. The low-resonance component, containing more impact fault information, was selected for the subsequent feature-extraction operation, as shown in Figure 26a,b.

**Figure 27:**
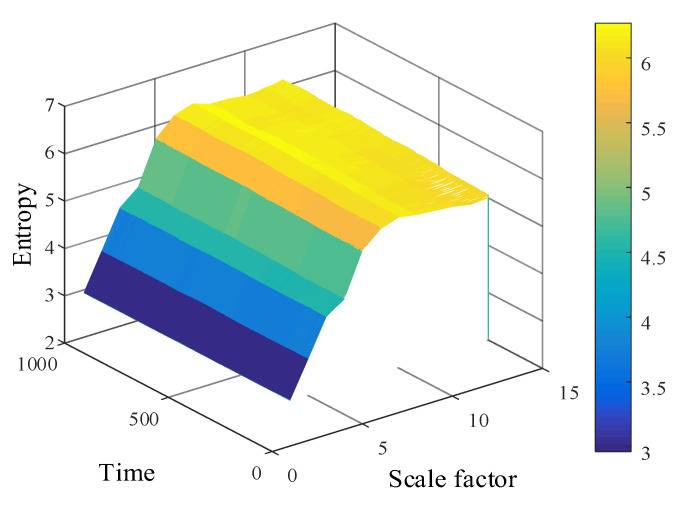
Multiscale permutation entropy feature matrix of No. 3 bearing.

**Figure 28:**
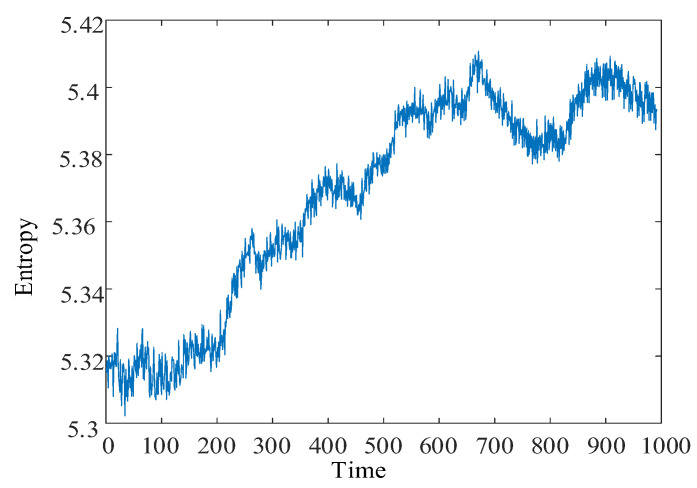
No. 3 bearing: MFPE feature.

After decomposing to obtain low-resonance components, the calculation of multiscale permutation entropy was performed. The multiscale permutation entropy feature matrix was obtained, as shown in Figure 27. The LLE was used to reduce and fuse the high-dimensional entropy matrix to obtain multiscale fused permutation entropy values, as shown in Figure 28.

**Figure 29:**
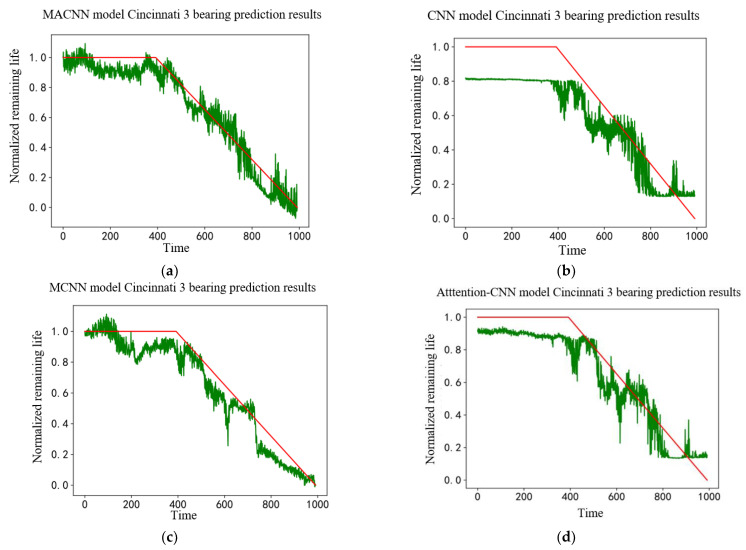
Prediction results of different models, (**a**) MACNN model prediction results; (**b**) CNN model prediction results; (**c**) MCNN model prediction results; (**d**) attention–CNN model prediction results.

The MACNN-based bearing life model was built, and the MFPE feature matrix size was reconstructed to (992, 1, 12) to meet the model input and training needs. MFPE features were input into the built MACNN life prediction model, and the prediction results were obtained, as shown in Figure 29a. The prediction results of the CNN model, MCNN model, and attention–CNN model are shown in Figure 29b–d, respectively. Since the bearing life was not given in the Cincinnati dataset, the normalized remaining useful life was used to represent the remaining useful life.

The prediction results shown in Figure 29 show that the overall life prediction performed by the MACNN model was better. The general trend was the same as the actual life curve, and the life prediction value deviated less from the actual value. Compared with the MACNN life prediction model, the MCNN model deviated from the predicted trend at the end of the bearing life, and the overall model deviated from the actual value. The CNN model and attention–CNN model failed to reflect the actual bearing life trend at the end of the bearing life, and the overall life prediction value deviated from the actual value. The life prediction result comparison between different models showed that the overall performance of the CNN model was improved by adding the multiscale feature-extraction module. The attention mechanism can bring the life prediction results closer to the actual values and improve the accuracy of life prediction.

**Figure 30:**
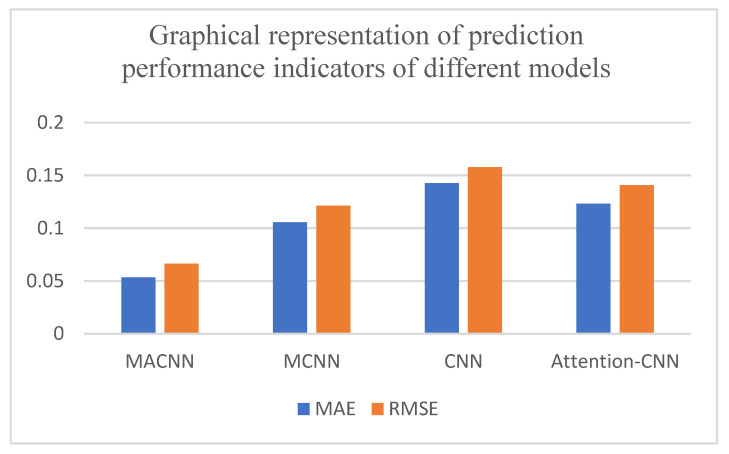
Graphical representation of prediction performance indicators of the different models.

Three metrics, MAE, RMSE, and model score, were used to quantitatively analyze the model prediction results, as shown in Table 3 and Figure 30. It can be seen that the MACNN model improved by 12.47%, 39.07%, and 22.54% in total score compared with the MCNN model, CNN model, and attention–CNN model, respectively. It shows that the MACNN model had better prediction accuracy and comprehensive performance in life prediction. The MAE evaluation index and the MSE evaluation index were reduced by 45.31%, 57.94%, and 52.86% compared with those of the MCNN model, the CNN model, and the attention–CNN model. These data show that the MACNN model has better generalization ability.

### 4.3. XJTU-SY Bearing Data Validation

**Figure 31:**
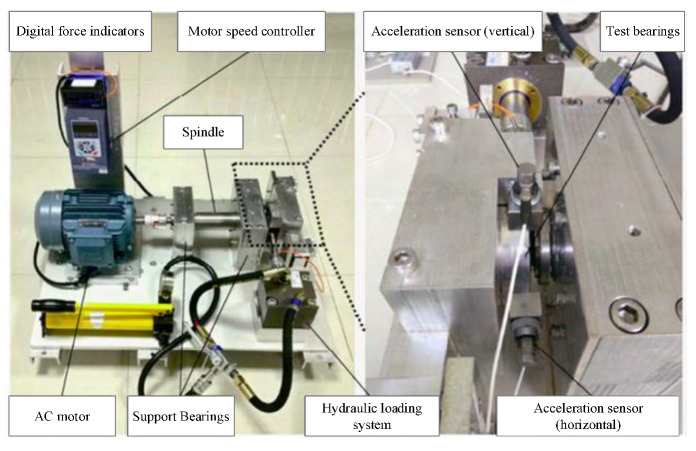
Data acquisition lab bench.

The XJTU-SY bearing dataset [36] was used for the experimental validation. This empirical dataset was collected by the empirical bench shown in Figure 31. The empirical bench mainly contained a motor speed controller, an acceleration sensor, a hydraulic loading system, the AC motor, and other components. The experimental bench can simulate various working conditions by adjusting the load and the speed.

**Figure 32:**
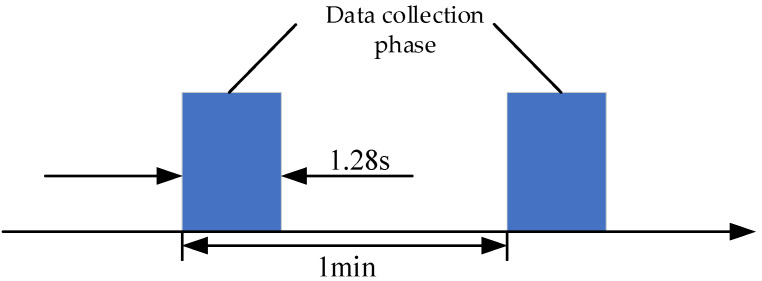
Vibration signal sampling settings.

As shown in Figure 32, in the experiment, the bearing data were collected by the acceleration sensors in horizontal and vertical directions. Two PCB 352C33 accelerometers were positioned at 90° to monitor the degradation of the bearings. The sampling frequency was set to 25.6 kHz, the sampling interval was set to 1 min, and the duration of each sampling was 1.28 s.

**Figure 33:**
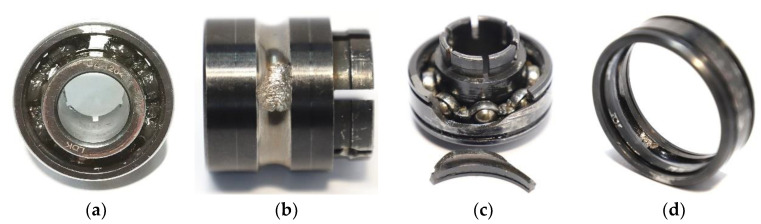
Experimental bearing failure categories: (**a**) cage fracture; (**b**) inner ring wear; (**c**) cracked outer ring; (**d**) outer ring wear.

The experimental bearings were LDK UER204 rolling bearings, whose relevant parameters are shown in Table 4. The failure locations were labeled on different bearings. The experiments were designed with three types of working conditions, containing four types of faults, as shown in Figure 33, with five bearings under each kind of working condition. The specific experimental data are shown in Table 5. Bearing1_1 data were used as the model validation set, and Bearing1_2 data were used as the model training set in the experimental validation.

**Figure 34:**
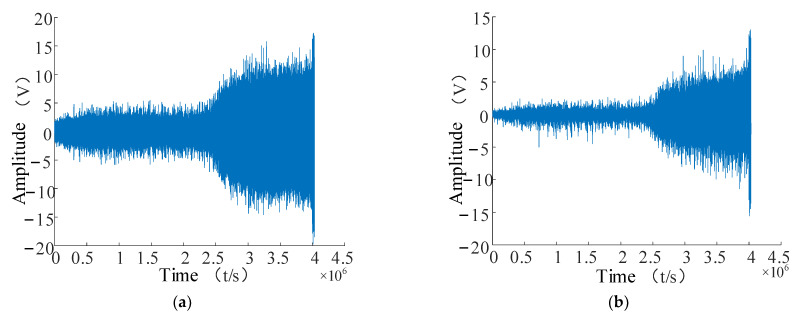
Bearing1_1 resonant sparse decomposition results: (**a**) Bearing1_1 high-resonance fraction; (**b**) Bearing1_1 low-resonance component.

The feature-extraction operation was performed on the collected data. Firstly, the data resonance sparse decomposition was processed, as shown in Figure 34a,b.

**Figure 35:**
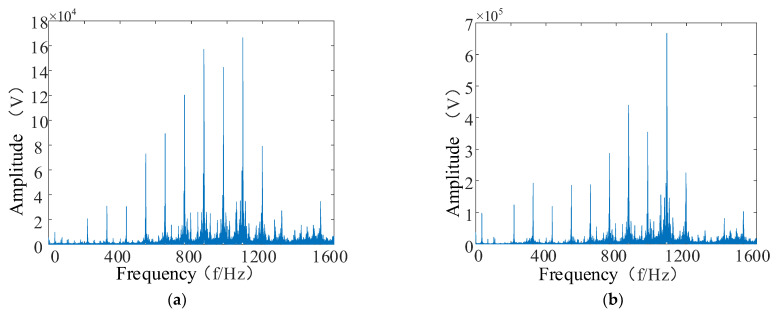
Comparative analysis of envelope spectra. (**a**) Low-resonance component envelope spectrum; (**b**) original signal envelope spectrum.

From Figure 35a,b, it can be seen that the characteristic information in the low-resonance component is more prominent. Although the overall amplitude of the low-resonance components obtained after the resonance sparse decomposition decreased, the feature information in the signal was more prominent. The ratio of frequency components from the 200 Hz–1000 Hz frequency interval to the highest frequency component became smaller, and the feature information in these frequency bands can be better extracted.

**Figure 36:**
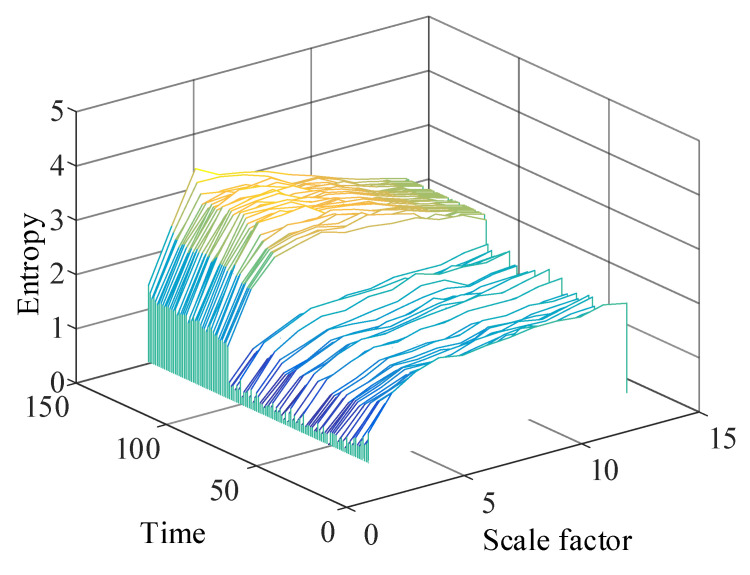
Multiscale permutation entropy matrix.

The low-resonance component of the resonance sparse decomposition was selected, and the multiscale permutation entropy value of the low-resonance component was calculated. The multiscale permutation entropy matrix was obtained, as shown in Figure 36. As the remaining useful life of the bearing declined, the multiscale permutation entropy value showed a significant upward trend. Additionally, the corresponding amplitude in the bearing vibration signal rose stepwise. This reflects the occurrence of severe bearing degradation well.

**Figure 37:**
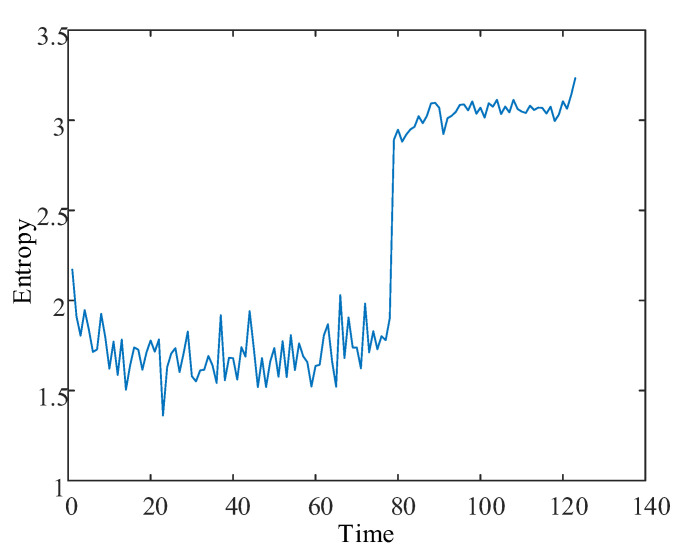
MFPE feature curve.

MFPE features were obtained by fusing the high-dimensional entropy matrix using the LLE dimensionality reduction algorithm, as shown in Figure 37. MFPE features can reduce the feature redundancy while retaining the primary remaining useful life trend information in the multiscale permutation entropy matrix. The same step-up in the MFPE features occurred when the bearing was severely degraded.

**Figure 38:**
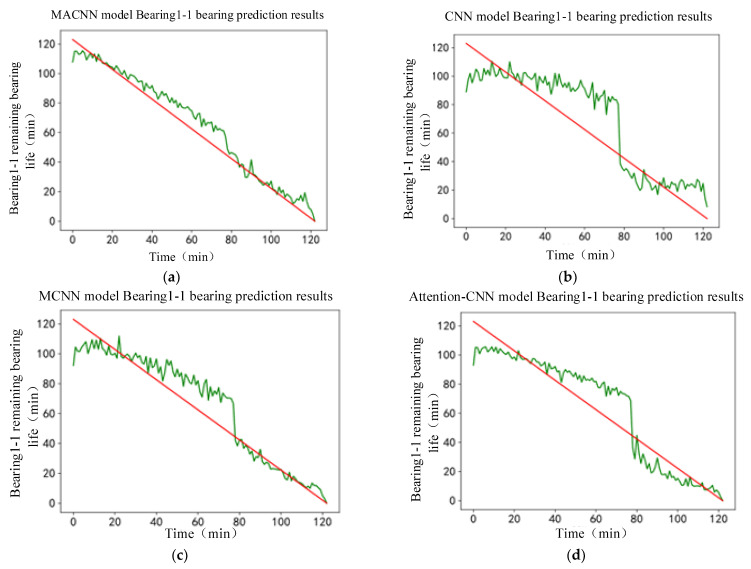
Prediction results of remaining useful life of bearings for different models: (**a**) MACNN model prediction results; (**b**) CNN model prediction results; (**c**) MCNN model prediction results; (**d**) attention–CNN model prediction results.

The MACNN-based bearing life prediction model was constructed, and the MFPE feature matrix size was reconstructed to (992, 1, 12) to meet the model input and training needs. The MFPE features were input into the MACNN life prediction model, and the prediction results were obtained as shown in Figure 38a. The prediction results of the CNN model, the MCNN model, and the attention–CNN model are shown in Figure 38b–d, respectively.

From the life prediction results, it can be seen that the general life prediction of the MACNN model was better. The overall trend was the same as the actual life curve, and the life prediction value deviated less from the actual value. Compared with the MACNN life prediction model, the MCNN model deviated from the predicted trend at the end of the bearing’s life, and the overall variation of the model from the actual value was more significant. The CNN model and the attention–CNN model failed to reflect the actual bearing life trend at the end of the bearing’s life, and the overall life prediction value deviated from the actual value. A comparison of the life prediction results between the different models showed that the overall performance of the CNN model improved with the addition of the multiscale feature-extraction module. The attention mechanism can bring life prediction results closer to the real values and improve the accuracy of the life prediction.

**Figure 39:**
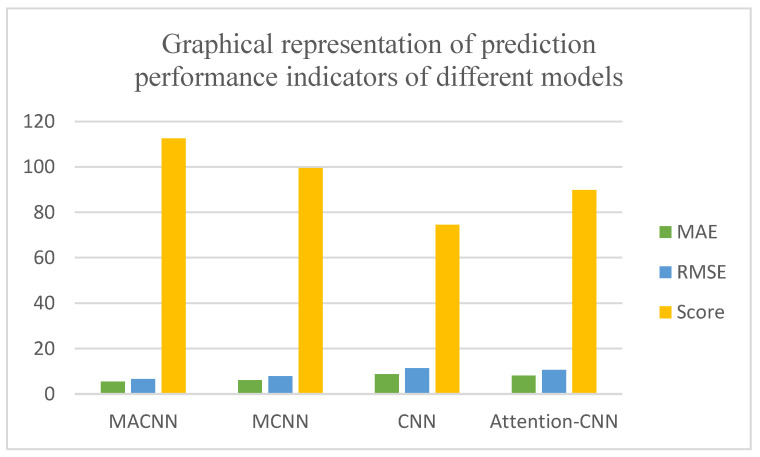
Graphical representation of prediction performance indicators of different models.

Three metrics, MAE, RMSE, and model score, were used to quantitatively analyze the models’ prediction results, as shown in Table 6 and Figure 39. From Table 6, it can be seen that the MACNN model improved by 13.17%, 51.01%, and 25.36% in total score compared with the MCNN model, the CNN model, and the attention–CNN model, respectively. These data show that the MACNN model has better accuracy and comprehensive performance in predictions of remaining useful life. Compared with the MCNN model, the CNN model, and the attention–CNN model, the MAE evaluation index was reduced by 9.91%, 37.41%, and 32.5%, respectively. These data show that the MACNN model has a better fitting ability. In the RMSE evaluation index, it was reduced by 15.03%, 41.98%, and 38.02% compared with the MCNN model, the CNN model, and the attention–CNN model, respectively. These data show that the MACNN model has better generalization ability.

To further demonstrate the effectiveness of the proposed method, the method was compared and validated with different RUL methods from three studies. The specific results are shown in Table 7.

As presented in Table 7, a fused CNN-based method for predicting the remaining life of rolling bearings was proposed in [37], and the MAE was 8.5176 by studying the accelerated life dataset from a test of XJTU-SY rolling bearings. This study used the MFPE combined with resonant sparse decomposition, then used the MACNN prediction model for the remaining useful life, and the MAE was 5.47256. Compared with other methods, the MFPE–MACNN model has improved fitting ability and prediction accuracy.

## 5. Conclusions

In this paper, an MFPE–MACNN model was proposed for the prediction of the remaining useful life of rolling bearings. This study solved the problem posed by the fact that the convolution-based deep learning model complicates the extraction of feature information from complex time series. The problem of redundant information concerning rolling bearing recession features was removed. The prediction accuracy of the rolling bearing life was improved. The multiscale fusion permutation entropy-based feature-extraction method extracts the MFPE features with low-resonance components, quantifies the evaluation signal time complexity, and reflects the decline trend of the remaining useful life. The remaining useful life prediction model for rolling bearings, based on the multiscale convolutional attention neural network, can extract the feature information of MFPE features at different time scales, fuse multiscale features, improve the fitting ability of the model, and reduce the prediction bias. The XTJU-SY rolling bearing complete lifecycle dataset was used for experimental validation and compared with other remaining useful life prediction models. Compared with the MCNN model, the CNN model, and the attention–CNN model, the MAE evaluation index was reduced by 9.91%, 37.41%, and 32.5%, respectively. The RMSE evaluation index was reduced by 15.03%, 41.98%, and 38.02% compared with the MCNN model, the CNN model, and the attention–CNN model, respectively, indicating that the MACNN model has improved fitting ability and generalization ability. The prediction error of the MACNN model occurs within 5 min, which means that researchers can better capture the information of life decline characteristics, with suitable accuracy for remaining useful life prediction.

The limitation of this article is that the overall effect of the proposed feature extraction fluctuates wildly when the bearing operating conditions are more complex, which may lead to significant deviations in the prediction of subsequent remaining useful life prediction models. In future research, a more stable feature-extraction method will be investigated to evaluate the remaining useful life of rolling bearings. Another shortcoming is that the proposed prediction model for the remaining useful life of rolling bearings has more training parameters and the model training time is longer. The model needs to be retrained after changing the bearing type or working conditions. In future research, the migration learning method will be used to solve this problem, and to improve the overall generalization and prediction efficiency of the model.

## Figures and Tables

**Table 1:** MACNN structural parameters.

Layer Name	Convolution Kernel Size	Number of Convolution Kernels	Layer Name	Convolution Kernel Size
Convolutional layer 1	1 × 1	1	Convolutional layer 3–2	2 × 2
Convolutional layer 2–1	2 × 2	1	Convolutional layer 3–3	3 × 3
Convolutional layer 2–2	3 × 3	1	Convolutional layer 3–4	4 × 4
Convolutional layer 2–3	4 × 4	1	Convolutional layer 3–5	5 × 5
Convolutional layer 2–4	5 × 5	1		

**Table 2:** Experimental validation of model parameter settings.

Parameter Name	Parameter Value	Parameter Name	Parameter Value
Input layer size	(1, 12)	Loss function	MSE
Output layer size	(1, 4096)	Optimizer	Adam
Training set size	(123, 1, 12)	Dropout layers	1
Test Set Size	(256, 1, 12)	Number of training sessions	100
Number of hidden layers	1	Batch size	12
Learning Rate	0.005	Activation function	ReLU

**Table 3:** Predictive performance metrics for different models.

Models	MAE	RMSE	Score
MACNN	0.05361817	0.06643362	102.16
MCNN	0.10571001	0.12147898	90.83
CNN	0.14271978	0.15796721	73.46
Attention–CNN	0.12339631	0.14092635	83.37

**Table 4:** LDK UER204 bearing parameters.

Parameter Name	Numerical Value	Parameter Name	Numerical Value
Inner ring raceway diameter—mm	29.30	Ball diameter—mm	7.92
Outer ring raceway diameter—mm	39.80	Number of balls	8
Bearing mid diameter—mm	34.55	Contact angle—(°)	0
Basic dynamic load rating—N	12820	Basic static load rating—KN	6.65

**Table 5:** XJTU-SY bearing data information list.

Work Conditions	Dataset	Total Number of Samples	L10	Actual Life Span
1	Bearing1_1	123	5.600~9.677 h	2 h 3 min
	Bearing1_2	161		2 h 41 min
	Bearing1_3	158		2 h 38 min
	Bearing1_4	122		2 h 2 min
	Bearing1_5	52		52 min
2	Bearing2_1	491	6.786~11.726 h	8 h 11 min
	Bearing2_2	161		2 h 41 min
	Bearing2_3	533		8 h 53 min
	Bearing2_4	42		42 min
	Bearing2_5	339		5 h 39 min
3	Bearing3_1	2538	8.468~14.632 h	42 h 18 min
	Bearing3_2	2496		41 h 36 min
	Bearing3_3	371	data	6 h 11 min
	Bearing3_4	1515	data	25 h 15 min
	Bearing3_5	114	data	1 h 54 min

**Table 6:** Predictive performance metrics for different models.

Models	MAE	RMSE	Score
MACNN	5.47256352	6.60530615	112.56
MCNN	6.07436166	7.77735746	99.46
CNN	8.74338361	11.38403952	74.54
Attention–CNN	8.10761687	10.65662429	89.79

**Table 7:** Comparison of different RUL models.

Author	Method	MAE
This paper	MFPE−MACNN	5.47256
Xiaodong Yang et al. [37]	Fusion–CNN	8.5176

## Data Availability

Not applicable.
